# Dental Students' Confidence and Competency in Root Canal Treatment Across Different Phases of the Dental‐Program: A Prospective Cohort Study

**DOI:** 10.1111/eje.70026

**Published:** 2025-07-29

**Authors:** Gabriela Umpierre Crespo, Renata Grazziotin, Patrícia Santos da Silva, Camila Hélen Grock, Francisco Montagner

**Affiliations:** ^1^ Curso De Odontologia Universidade Do Vale Do Taquari Lajeado Brazil; ^2^ Faculty of Dentistry University of British Columbia Vancouver British Columbia Canada; ^3^ Faculdades Do CEFI Porto Alegre Brazil; ^4^ Private Practice Limited to Endodontics Erechim Brazil; ^5^ Departamento De Odontologia Conservadora, Faculdade De Odontologia Universidade Federal Do Rio Grande Do Sul Porto Alegre Brazil

**Keywords:** clinical care, competence, confidence, dental students, preclinical training, root canal treatment

## Abstract

**Introduction:**

This prospective cohort study evaluated dental students' confidence and competency in root canal treatment across their dental programme, encompassing the transition from preclinical laboratory to clinical patient care.

**Material and Methods:**

A cohort of 23 dental students was assessed during four phases of the DDS program over three academic semesters (18‐month calendar year). Data collection occurred at the following stages: upon completion of the preclinical endodontic laboratory course (P1); at the conclusion of the Comprehensive Clinical Care I course, where students performed single‐rooted canal treatments on patients (P2); after training with plastic/artificial multi‐rooted teeth (P3); and upon completion of the Comprehensive Clinical Care II course, where students performed multi‐rooted canal treatments on patients (P4). Self‐reported confidence levels were measured using Likert scales, while root canal treatment competency was evaluated based on final radiographic outcomes. Focus group discussions explored students' perceptions of self‐confidence and competence across the four phases and during the transition from preclinical to clinical practice. Quantitative data were analysed using statistical methods, while qualitative data were examined through thematic analysis.

**Results and Discussion:**

Confidence levels increased from P1 to P2, reaching a peak at P3 and P4. Participants demonstrated higher technical competency in root canal obturations during patient care than in laboratory work. Key themes were expectations/feelings and technique, with pulp chamber access opening identified as the most challenging procedure. The transition to clinical care induced fear, anxiety, and insecurity initially, but participants reported reduced anxiety and improved technical performance when working with patients.

**Conclusion:**

Dental students exhibited enhanced competence and confidence in endodontic treatments when performed in the clinical setting, reflecting positive progression across the evaluated requirements.

## 1 Introduction

Dental education has been described as one of the most challenging fields of study and one of the most demanding and stressful areas of healthcare [1]. Dental training involves integrating knowledge and skills in different scenarios, from technical topics to interpersonal subjects [2, 3]. In endodontics/root canal treatment, dental schools usually separate the teaching into preclinical laboratory training (in which students perform procedures in mannequins, plastic teeth and human extracted teeth) and clinical care (where students provide dental care for patients). The acquisition and construction of knowledge in dentistry face challenges that extend beyond the mere availability of information. While most studies suggest that additional education is needed to bridge knowledge gaps, no direct evidence supports the notion that a lack of education is their root cause [4]. Clinical practice often prioritises established protocols and standard procedures over continuous evidence‐based updates [4]. Therefore, it is crucial to reassess educational strategies to ensure that knowledge acquired during training is effectively translated into technical competence and evidence‐based decision‐making.

Acquiring competencies in undergraduate courses in Endodontics comprises the development of knowledge, skills, attitudes, and professionalism. It can be complex not only due to the technical difficulties involved in the procedures but also due to the responsibilities inherent to patient care and the students' lack of self‐confidence [5, 6, 7]. In most dental programmes of our region, the competency‐based approach has replaced the traditional assessment methods (number of procedures) in clinical practice. Competency‐based means that the student's understanding, psychomotor skills, professional values and personal confidence are essential factors in starting the unsupervised practice of dentistry [8]. The cognitive and psychomotor competencies should ideally be continuously evaluated through self‐assessment by the dental professional, considering their knowledge and skills. ESE guidelines for dental training indicate that students should be competent at performing root canal treatment on uncomplicated anterior and posterior teeth [9]. Recently, the American Association of Endodontists has provided recommendations on which clinical cases can be managed by less experienced practitioners, and which should be referred to specialists, contributing to a critical appraisal of confident and evidence‐based practice among dentists [10].

Although competency is fundamental to dental practice, confidence plays a crucial role in its development and should not be underestimated. The quality of education and clinical experience are key factors in shaping both dental students' self‐perceived confidence and overall competence [11, 12]. In endodontic treatment, particularly, competence in performing specific procedural steps appears to be directly influenced by students' exposure to these techniques, reinforcing the importance of hands‐on training in building both skill and confidence [13]. The transition from theory to practice in health education is reported as an emotional and socially dynamic process. While this transition to clinical training can be a time of significant personal and professional development, it can also be a source of stress and anxiety [5]. In most dental programmes, the transition stage from preclinical to clinical setting occurs within a short period (a few weeks). Then, during this period, dental students must adapt cognitive and psychosocial skills to treat patients. The practical skills they learned during preclinical education must be readily translated to function successfully in the clinical setting [6, 14, 15].

In most dental programmes of our region, the competency‐based approach has replaced the traditional assessment methods (number of procedures) in clinical practice. Competency‐based means that the student's understanding, psychomotor skills, professional values and personal confidence are essential factors in starting the unsupervised practice of dentistry [8].

Understanding the progression of competencies and experiences of undergraduate dental students during the transition from the preclinical to the clinical phase in Endodontics is essential. However, few longitudinal studies have tracked the same group of students throughout their training. Continuous assessment of this process can provide valuable insights for improving teaching methods and enhancing the education of future dentists, effectively preparing them for professional practice. Therefore, this study hypothesises that investigating dental students' confidence in performing procedures [13, 16] and their competency development would provide a valuable approach to better understanding the teaching‐learning process, particularly in the field of Endodontics, which remains underexplored in the literature [17, 18].

This prospective cohort study aimed to assess dental students' confidence and competency in root canal treatment using quali‐quanti data collection and analysis during four different phases of the dental programme, including the transition from preclinical laboratory to clinical patient care.

## 2 Material and Methods

### 2.1 Study Design and Ethics

A longitudinal prospective study was conducted in a public dental school in southern Brazil, following a cohort of dental students over three academic semesters (approximately 18 months in the calendar year). During this period, dental students underwent four phases of their dental programme (DDS degree). The ethical review board approved the project (approval number protocol CAAE 89051118.4.0000.5347).

### 2.2 Participants Recruitment and Inclusion Criteria

All 35 students in the dentistry cohort enrolled in the preclinical endodontic laboratory course were invited to participate, as they represented the entire group at this stage of the program (convenience sample). The student was included in the study if they were 18+ years old and agreed to participate. All the participants signed an informed consent before joining the study.

### 2.3 Context

The study cohort was followed throughout the extended preclinical endodontic lab, comprehensive clinical care I, the short preclinical endodontic lab, and comprehensive clinical care II courses. The preclinical endodontic lab course (6 months) takes place in the second year of the dental programme and consists of lectures (10 h) and practical training (52 h). The lectures cover essential technical topics and fundamental endodontic principles, including access cavity preparation, cleaning and shaping, and root canal obturation. Practical training is conducted on artificial teeth/plastic models (one anterior tooth, one premolar, and two molars).

The Comprehensive Clinical Care I course (6 months, 255 h of practical training) takes place in the third year of the dental programme, where students provide dental care for patients requiring non‐complex clinical procedures.

The Comprehensive Clinical Care II course (6 months, 225 h of practical training) also takes place in the third year of the dental programme. Initially, they undergo a new, short preclinical lab course—lasting 9 h—focused on endodontic treatment of molars using artificial teeth before advancing to more complex clinical activities. Subsequently, they provide emergency dental care for patients and treat the root canals of at least one multi‐rooted tooth.

### 2.4 Instruments and Period of Data Collection

Data was collected at four distinct points during the DDS programme, as follows: Assessment Point 1 (P1)—at the end of the preclinical endodontic lab course; Assessment Point 2 (P2)—at the end of the comprehensive clinical care I course; Assessment Point 3 (P3)—immediately after the short‐duration preclinical training in multi‐rooted teeth, just before the comprehensive clinical care II course; and Assessment Point 4 (P4)—at the end of the comprehensive clinical care II course.

Dental students' self‐reported confidence to perform root canal treatment procedures was collected using a short questionnaire containing Likert scales for each one of the following procedures: anaesthesia (Anest), rubber dam placement (RDP), access opening (AO), working length radiograph (WLR), cleaning–shaping (CS), intracanal medication placement (ICM), tug‐back test for the master gutta‐percha cone (O‐TB), cone fit radiograph (O‐Rx), obturation (O‐P) and temporary coronal restoration (TR). The 5‐point Likert scale included the following levels: ‘very little confident’, ‘little confident’, ‘neutral’, ‘confident’ and ‘very confident’.

Dental students' competency in performing root canal treatment with adequate technical quality was assessed using the final radiograph of obturated teeth. Adequate technical quality was determined based on obturation length, density, and taper [19]. The evaluation was conducted jointly by three researchers (G.U.C., C.H.G. and F.M.), who collectively assigned a single consensus score, as the assessment criteria were well‐defined and objective, minimising subjectivity. Length was categorised as follows: adequate—when the obturation was positioned 0.1–2 mm short of the radiographic apex; overfilling—when the obturation extended beyond the radiographic apex; underfilling—when it was more than 2 mm short of the radiographic apex; and at the limit—when the obturation coincided with the radiographic apex. Density was classified as adequate (1) when the obturation was uniform and free of voids, or inadequate (2) when empty spaces were present within the obturation or at the interface between the obturation and the canal walls. Taper was considered adequate (1) when the canal taper increased gradually from the crown to the apical foramen, or inadequate (2) when the taper was irregular or inconsistent. Although different criteria exist for evaluating the final radiograph of obturated teeth, the assessment was conducted with the expectation that students follow the standards taught in their curriculum.

Dental student' perceptions of self‐confidence and competence at four different moments of the dental programme, including during the transition from preclinical training to clinical care, were assessed in focus group discussions. There were 8 focus groups, being 2 in each one of the study assessment points. Participants were divided into two groups of nine individuals each. The allocation of participants into the group was based on block randomisation, that is, a mix of high achievers, average, and low achievers—considering their grades obtained in the preclinical endodontic lab course (beginning of this study). An individual with expertise in qualitative research led the focus groups. The discussions were recorded and immediately transcribed.

### 2.5 Data Analysis

Data originated from the investigations on (1) ‘self‐reported confidence to perform root canal treatment procedures’ and (2) ‘competency to produce a root canal treatment with adequate technical quality’ were statistically analysed. Comparison of ‘confidence’ between different endodontic procedures in the same assessment point was measured by Kruskal–Wallis and Dunn post hoc tests (*α* = 5%). Comparison of ‘confidence’ between the different assessment point of the study (same endodontic procedure) was measured by the Student *T* test (*α* = 5%). Comparison of ‘competence’ was measured similarly to the one used for confidence, with the Student *T* test (*α* = 5%) for length, density, and taper. Statistical analysis was performed using SPSS (IBM SPSS v.18.0, SPSS. Chicago, IL, USA). The Spearman test was conducted to determine the correlation between the competence criterion ‘quality of obturation’ and the self‐reported confidence in performing the steps of the tug‐back test for the master gutta‐percha cone (O‐TB), cone fit radiograph (O‐Rx) and obturation (O‐P). The competence criterion ‘length of obturation’ was correlated with O‐TB and O‐Rx, while the criteria ‘density of obturation’ and ‘taper of obturation’ were correlated with O‐P. Correlations were analysed separately for each Assessment Point.

Data originated from the eight focus groups (2 in each assessment point) were thematically analysed. The transcribed sentences were exported to NVivo (QSR International Pty Ltd. Version 12, 2018). Codes and sub‐codes were created based on the sentence content. The codes were quantified using graphs (NVivo) to compare the beginning of the endodontic training (P1 and P2) with the highest levels of endodontic training (P3 and P4). We constructed a word cloud to summarise all the words mentioned in all the focus groups. Larger words in the image represented the words more frequently mentioned by participants. Results were then descriptively reported and expressed as absolute or relative frequency, median, and 25th and 75th percentiles.

## 3 Results

Of the 35 invited students, 29 (*n* = 24 females, *n* = 5 males) agreed to participate, with an average age of 21 years (range: 19–27 years). Throughout different stages of their training, some participants were lost due to academic performance or personal reasons. Specifically, four students did not progress from the extended preclinical training to the clinical stage (three failed the course, and one withdrew), and two additional students were lost during the advanced clinical training stages (both failed the course). Consequently, by the end of the study, the final number of participants was 23.

Students became more confident with the onset of their clinical training. Participants had little confidence to perform root canal treatment procedures in P1 (preclinical training with single‐rooted teeth) for rubber dam placement, intracanal medication placement, and temporary coronal restoration.

**FIGURE 1:**
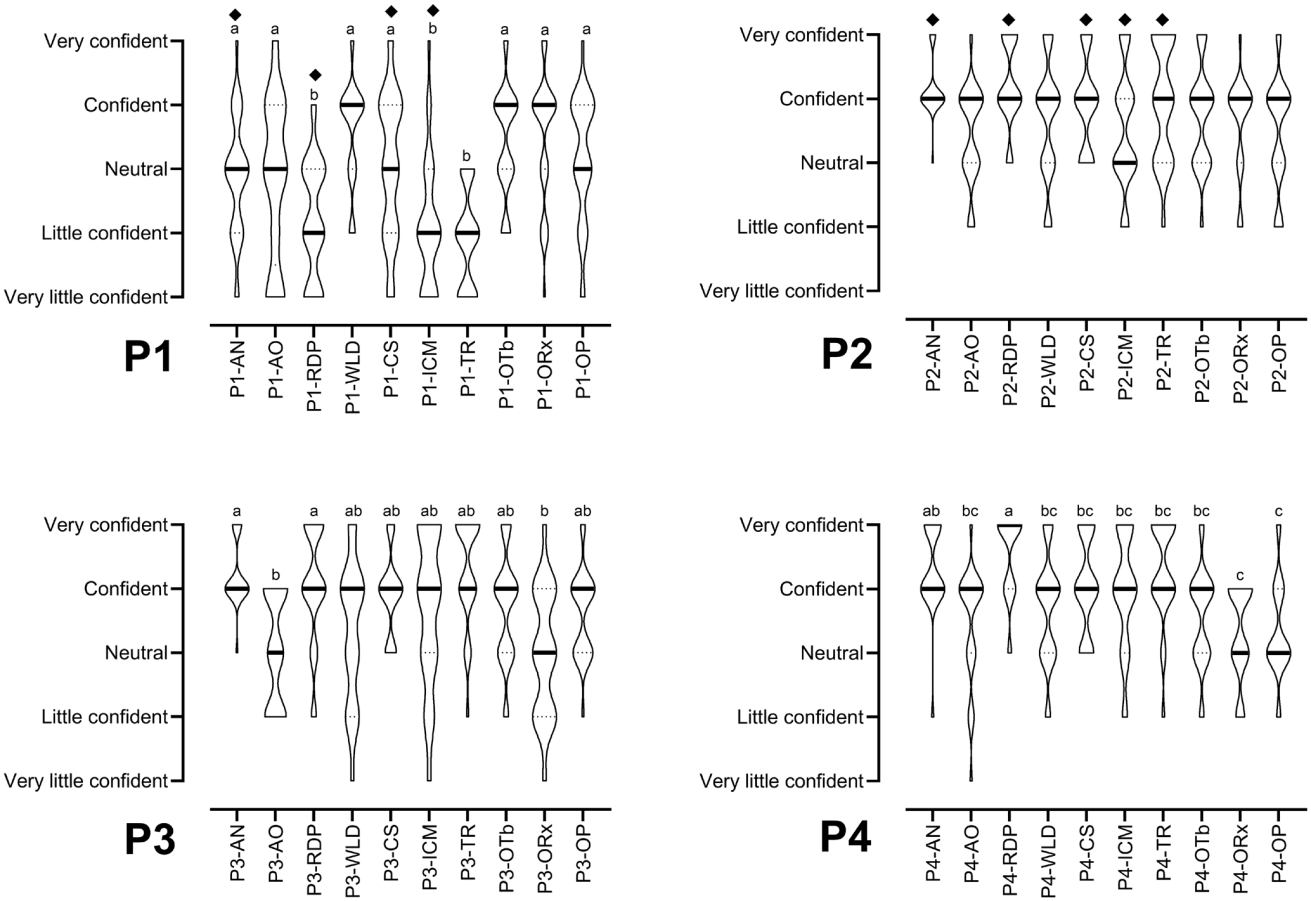
Confidence scores for performing endodontic treatments on single‐rooted teeth, considering different stages of the endodontic training. Equal lowercase letters indicate no statistically significant difference between stages in the same period (Kruskal–Wallis' test, *p* > 0.05). The symbol ◊ indicates a statistically significant difference between the same procedure stage at P1 versus P2, or P3 versus P4 (Student's *T*‐test, *p* < 0.05).

For the rest of the procedures, median values varied between ‘neutral’ and ‘confident’. Increased confidence levels were noted in P2 (comprehensive clinical care I course) in rubber dam placement, cleaning–shaping, intracanal medication placement, and temporary coronal restoration. Figure 1 shows that participants were ‘confident’ at P3 (immediately after plastic multi‐rooted teeth training, in the first half of the comprehensive clinical care II course) in most of the procedures, except in access opening and cone fit radiograph, where students felt ‘neutral’. A uniform distribution of participants concerning the scores indicated variation in confidence levels between participants in the different phases of the DDS programme/endodontic training. From P3 to P4, the median values for most of the procedures were maintained as ‘confident’. The only two procedures with increased confidence were rubber dam placement (from score ‘neutral’ to ‘confident’) and access opening (from score ‘low confidence’ to ‘neutral’).

**FIGURE 2:**
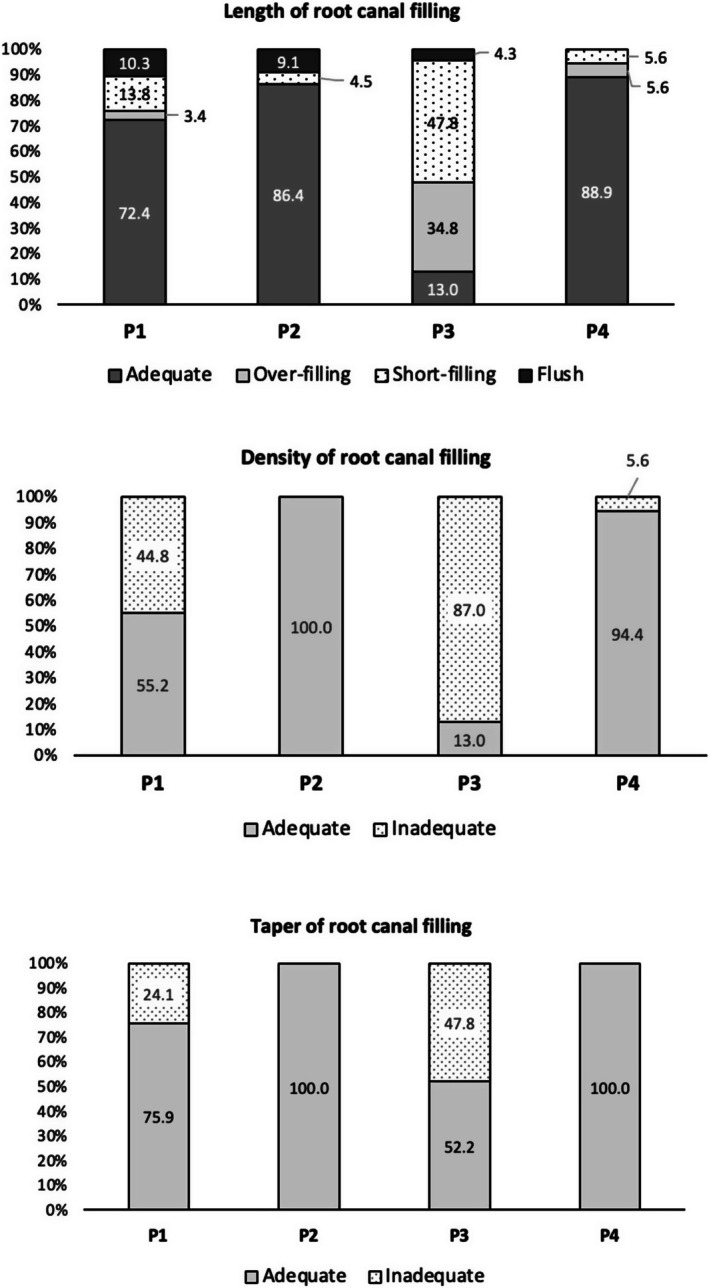
Radiographic quality of the root fillings, according to Balto et al. [19]

Dental students' competency in performing root canal treatment with adequate technical quality was assessed using the final radiograph of obturated teeth. Figure 2 shows that length was classified as ‘adequate’ in 70% and 80% of cases in P1 and P2, respectively (*p* > 0.05). From P3 to P4, ‘adequate’ obturations increased from 12% to 89% (*p* < 0.05). Density in the root canal filling showed a higher number of ‘adequate’ scores in clinical training stages (P2 and P4) compared to their respective preclinical training stages (P1 and P3) (*p*‐value P1 vs. P2 = 0.0002; and *p*‐value P3 vs. P4 = 0.0001). Regarding the variable ‘taper’, there were more ‘adequate’ obturations in the clinical training stages compared to the corresponding preclinical stages (*p*‐value P1 vs. P2 = 0.0150; and *p*‐value P3 vs. P4 = 0.0008).

No statistically significant correlation was found between the competence criteria for quality of obturation (length of obturation, density of obturation and taper of obturation) and the self‐reported confidence in performing the corresponding procedural steps (tug‐back test for the master gutta‐percha cone, cone fit radiograph and obturation) within the same phase. Additionally, when analysing the overall results, no statistically significant correlation was observed between the competence criteria related to quality of obturation and the self‐reported confidence in executing the same procedural steps.

**FIGURE 3:**
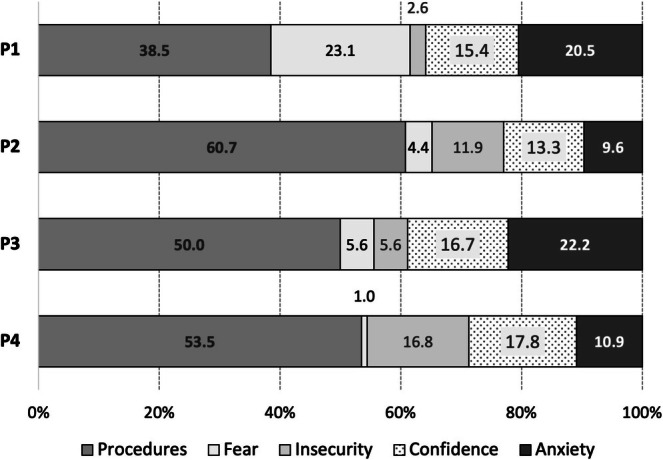
Matrix encoding graph: percentage of mentions by participants considering the analysis nodes at each moment of the study.

Dental students' perceptions of self‐confidence and competence at four distinct phases of the dental programme, including during the transition from preclinical training to clinical care, were evaluated. Figure 3 shows the quantification of codes using matrix graphs comparing the beginning of the endodontic training (Phase 1 and Phase 2) with the highest levels of endodontic training (Phase 3 and Phase 4). The resulting codes (and sub‐codes) were expectations, feelings (anxiety, fear, confidence and lack of confidence), and technique (anaesthesia, rubber dam placement, access opening, working length radiograph, cleaning‐shaping, intracanal medication placement, tug‐back test for the master gutta‐percha cone, cone fit radiograph, obturation and temporary coronal restoration).

Data from the thematic analysis showed students' perceptions during the transition from preclinical to clinical settings. The expectations of most students in study Phase 1 were to face several difficulties, considering the comments made by senior peers. Students found the preclinical endodontic lab very demanding at the beginning. The access opening was considered the most challenging procedure. Having all the teeth accessed in the same session received negative criticism.

I always hear my senior peers conducting clinic treatments saying that endodontics is difficult. They say they are doing an endo‐therapy, and they will not be able to finish it by the end of the course because they had to separate it into several appointments […] My God! Endo will be tough. (Student A1, Phase 1).

At the beginning of the course, I was terrified, very scared! After it ended, I got an A (highest score) in one tooth… I did not even expect it, so the beginning was difficult’ (Student A5, Phase 1).

Before working on the first single‐rooted teeth, we had no idea what the professor was talking about in class; it is very abstract. This first contact with Endo made me very anxious (Student B2, Phase 1).

Yeah, and there is also a session where we access all our teeth. And then, at the end, our entire treatment was graded. There was no more time to correct the other teeth because we have already opened them all together. (Student A7, Phase 1).

Starting to provide clinical care for patients needing root canal treatment (transition from Phase 1 to Phase 2) was considered a sudden change, which caused fear, anxiety, and insecurity. More responsibility was needed in a short time (one academic semester) than at the beginning. Participants believed that the difficulties in endodontics were more significant compared to the other dentistry specialties.

It seems that now they have thrown us into the dentistry. At the beginning, there was little [information on] teeth, much histology [contents], many things, and then, we suddenly fell into the world of dentistry. (Student A5, initial period of Phase 2).

I am afraid because I keep thinking that I am going to be there with the patient and the tooth access will be more difficult, the x‐ray will be more difficult, everything will be more difficult (Student A7, initial period of Phase 2).

I find endo[dontics] the most difficult specialty because it needs much practice to get confidence, and we do not see what we are doing. Other specialties are more visual [than endodontics]. It takes longer for you to be a good endodontist than to be good in another area. (Student B3, Phase 2).

At the end of study Phase 2, participants provided endodontic treatment on single‐rooted teeth for their patients. Endodontic access opening generated a lack of confidence in the participants. Students stated that they needed help transferring theoretical knowledge to practical activity and felt unconfident during several stages of root canal treatment. Conversely, a few students felt independent and found the clinical working easier and faster than the preclinical lab working.

I did not make [endodontic] access through the enamel and dentin; I just removed the temp material, and it was easier [than in the Lab]. I think it is because you can see what needs to be removed. However, I also do not feel confident about opening a tooth because I have not done it yet. (Student B1, Phase 2).

I was completely dependent on helping, and I also did not feel confident because I never did it alone. (Student A8, Phase 2).

I found it difficult because my master cone bent, and I could not fix it. I was nervous about it, so I had to take everything out and do it again. (Student B9, Phase 2)

I was very anxious because I could not see where to put the cones. I put the spacer, and when I was going to put the cone, I could not [see it]. (Student B5, Phase 2).

I found it more peaceful than in the preclinic lab. (Student B1, Phase 2).

The unanimous opinion was that the transition from the preclinical to clinical phase during practising complex treatments (Phase 3 to Phase 4) was smoother than the previous transition. However, after performing treatments on multi‐rooted teeth in patients, participants emphasised their difficulties in access, radiographs, and obturation.

There is much difference between natural and plastic teeth… the plastic ones sometimes come from the factory with a defect. Then, the person who got a good artificial tooth had a positive experience, but I got a horrible tooth. My experience was negative, and that makes me frustrated—maybe that is why my experience was distorted. I became less confident and more anxious to perform multi‐rooted treatment in patients. (Student B9, at the end of Phase 3).

I am still scared, anxious and so on…, but it is less intense, it seems like I need to be more careful. (Student A4, initial period of Phase 4).

I do not feel confident. I only performed one endo‐access in a maxillary tooth, so …. it was terrible, difficult, I was not sure what I was doing. (Student A3, Phase 4).

I remained the entire appointment to get the working length. Every time I needed to take an X‐ray, I was in even more agony. (Student B4, Phase 4).

## 4 Discussion

The present prospective cohort study followed a group of dental students in a DDS program in the South of Brazil during 4 phases of learning endodontics. The study lasted 18 months, in which the students attended preclinical and clinical phases to learn the treatment of single‐rooted teeth (P1 and P2) and multi‐rooted teeth (P3 and P4). Through a quali‐quantitative analysis, we investigated dental student' perceptions (addressing their self‐confidence in doing procedures and competence by assessing the technical quality of root canal obturation) during the transition from preclinical (laboratory) to clinical activities (patient care)—taking into consideration the degree of complexity of endodontic treatments. Each assessment point was intentionally aligned with key curricular milestones to monitor the development of students' self‐confidence over time. Therefore, the assessment points represented a cumulative educational experience, where earlier stages were expected to influence later outcomes. This interdependence is inherent to the longitudinal design and was expected. Several factors were identified as modulators of the transition from preclinical training to clinical care, including the first contact with the patient, the lack of opportunities for additional preclinical training, and the discrepancy between the virtual and actual environments (considering the technical aspects presented in class, for example).

Strengths of this study included the use of a quali‐quantitative analysis, allowing a broader and deeper understanding of data—according to pertinent previous authors [7, 13, 20]; and the assessment of students' competency in root canal treatment procedures using parameters that are established in the literature as representatives of the technical quality of obturations [19, 21, 22]. The qualitative analysis from this study showed variation/alternation in the participant' confidence and feelings. Upon analysing the most prevalent codes in the focus groups, we noted higher levels of anxiety and fear during the preclinical laboratory periods (P1 and P3), which decreased during the clinical care periods (P2 and P4). The quantitative assessment of competence found more appropriate root canal obturations in the clinical care period, meaning that students were more competent in treating patients than treating artificial (and extracted human) teeth. Different behavioural, emotional, and technical issues were present in the student' perceptions of the transition stages (preclinical laboratory to clinical care). It occurred either in less complex (root canal treatment of single‐rooted teeth) or in more complex activities (root canal treatment of multi‐rooted teeth).

Perceptions emerging after initial preclinical training (P1) included a lack of confidence in performing specific procedures that had not been covered in the previous preclinical activities—the so‐called ‘unknown’ procedures—such as rubber dam placement, intracanal medication insertion, and temporary tooth restoration. A previous study also showed that unfamiliar procedures triggered negative feelings in clinical practice [7]. Considering these findings, we hypothesised that incorporating additional opportunities to teach preclinical students about unknown procedures could help bridge the gap between the ‘virtual’ and ‘real’ environments. In other words, theoretical instruction would be reinforced by hands‐on activities, providing a more comprehensive learning experience. By the end of the Comprehensive Clinical Care I course (P2), there was a significant increase in students' confidence scores regarding the unknown procedures. For instance, rubber dam isolation was mentioned as a procedure that no longer caused much concern, as students had several opportunities to practice it in other disciplines, such as restorative procedures and paediatric dentistry. This finding reinforces the importance of integrating disciplines and practices, even during the preclinical phases of the dental program [7, 23].

In preclinical activities, the endodontic access opening was considered one of the most challenging procedures. Participants pointed out the following challenges in access openings: the use of artificial teeth in the preclinical laboratory (P1 and P3), which did not replicate very well the human extracted teeth, and the lack of opportunity to access single‐rooted and multi‐rooted teeth in clinical practice (P2 and P4), since some patients have had the tooth already opened by another dentist (outside the university clinic, in an emergency service, for example). Previous authors have shown that dental students are open to practising on plastic teeth. However, the replicas available in the market are still deficient and would need improved radiopacity, anatomy, and material hardness [13, 23, 24].

For certain procedures, such as intracanal medication placement (P2 and P3) and cone fit radiograph (P1, P2, P3), the ‘neutral’ score predominated in self‐confidence assessments. This raises questions about the practical meaning of this response. According to Nadler et al. (2014) [25], who examined how participants interpret the midpoint labelled as ‘neither’, the most common interpretation was ‘no opinion’, followed by ‘don't care’, ‘unsure’, ‘neutral’, ‘equal/both’ and ‘neither’. Additionally, participants may select the midpoint when they are confused by the item. However, since these tasks are part of their practical routine, confusion should not be a likely reason for this choice.

The increase in students' confidence after beginning clinical practice was likely driven by their exposure to real patient care and the consolidation of their clinical skills. Participants reported feeling less anxious and achieving more technically appropriate root canal obturations when working with patients. Chambers [26] indicated that there is no standard to be followed to establish the number of previous experiences to which students should be exposed and which could favour clinical practice. Participants gain confidence in performing endodontic treatment steps as they face new and more complex experiences, reinforcing the idea that skill and competency development is cumulative and relies on the transition from preclinical to clinical training. Therefore, performing endodontic treatments in a clinical setting is crucial.

It is interesting to reemphasise that the participants produced more technically appropriate root canal obturations when working with patients (assessment of competency based on the quality of root canal obturations). This outcome may be associated with the learning progress and the clinical instructor's demonstration of procedures in real time with the dental patient (perception arose during focus groups). The teaching model in the dental school of this study is to leave the student more independent during the preclinical training and supervise more closely during clinical practice (i.e., the instructor frequently sits on the chair and performs the procedure for the student as a demonstration). The instructor's clinical assistance significantly increased students' competence in this current study.

In the present study, no significant correlation was found between self‐reported confidence and competence parameters in endodontic treatment procedures. Perceptions of competency in practical skills may differ between students and teachers. Stacey et al. (1998) [27] investigated the effect of clinical targets on clinical productivity and perceptions of competency among final‐year undergraduate dental students, comparing their self‐assessments to those of clinical teachers. By the end of the study, clinical teachers consistently scored students significantly lower in clinical competency than the students scored themselves, regardless of group assignment. Similarly, Tuncer et al. (2015) [28] found that lower‐performing students tended to overrate their performance, whereas higher‐performing students tended to underrate theirs during their second year of dental education. Educational research evaluating student learning should prioritise competency‐based assessments over student perceptions as the primary outcome measure [29]. However, self‐evaluation remains an essential skill for dental professionals, as they must develop evaluative criteria that will enable them to continually enhance their competence throughout their careers [28].

The results of the present study may also be influenced by the teaching practices adopted by the dental school. At the dental school where this study was conducted, preclinical activities were integrated into the curriculum twice, including one instance during the clinical training period. Kapler et al. (2019) [30] reported that the distribution of preclinical activities in Brazilian dental schools is highly heterogeneous, typically occurring between the third and tenth semesters, following the completion of basic science courses. Similarly, Al Raisi et al. (2019) [31] identified significant variability in the timing of preclinical training among UK dental schools. According to their study, 40% of institutions offer preclinical training during the second and third years, while 20% schedule it for the third and fourth years. Additionally, 13% provide training from the second to the fourth year, another 13% extend it from the second to the fifth year, and only 7% restrict preclinical training to the second year. Therefore, a standardised structure for endodontic teaching practices should not be expected, as it depends on the overall undergraduate curriculum, epidemiological factors, regional diversity, and cultural differences.

It could be hypothesised that multiple exposures to preclinical activities constitute an effective curricular strategy to reinforce the connection between preclinical training and clinical practice. Consistent with this, the present study and previous findings by Grock et al. (2019) [7] indicate that students frequently express the need for more intensive preclinical training. Limited exposure to preclinical and clinical Endodontics may lead to low self‐confidence during patient care, resulting in dissatisfaction and insecurity. Participants appreciated the opportunity to revisit preclinical exercises using extracted or plastic molars before transitioning to clinical practice. They valued the opportunity to reinforce their skills and confidence rather than focusing on the number of procedures performed. The results suggest that academic teaching strategies need to be dynamic, allowing for variations in participants' confidence and emotions. In this context, alternating preclinical and clinical training periods may serve as an example of a dynamic approach, as it helps mitigate negative emotions—preventing the buildup of apprehension and anxiety that students often experience while waiting to treat patients, a situation that in some schools occurs only at the end of the dental programme.

## 5 Conclusions

The first contact with the patient, the lack of opportunities to perform additional preclinical practice in plastic (or extracted) teeth, and the incompatibility between the ‘virtual’ and the ‘real’ environment (lectures and hands‐on) were the main factors modulating the transition from the preclinical laboratory training to the clinical care in endodontics. Endodontic access opening was the procedure that students had low confidence to perform in all the study periods—and this was associated with little exposure to clinical experience (not enough opportunities). The students became more competent and confident within the evaluated requirements when they performed endodontic treatments during the clinical setting (patients' care). The present study provides valuable insights, as similar research inquiries could be explored by other authors in different contexts, enabling the comparison of results across various educational settings.

## Conflicts of Interest

The authors declare no conflicts of interest.

## Data Availability

The data that support the findings of this study are available on request from the corresponding author. The data are not publicly available due to privacy or ethical restrictions.

## References

[1] M. Botelho , X. Gao , and S. Y. Bhuyan , “An Analysis of Clinical Transition Stresses Experienced by Dental Students: A Qualitative Methods Approach,” European Journal of Dental Education 22, no. 3 (2018): e564–e572. 29665214 10.1111/eje.12353

[2] J. Cowpe , A. Plasschaert , W. Harzer , H. Vinkka‐Puhakka , and A. D. Walmsley , “Profile and Competences for the Graduating European Dentist—Update 2009,” European Journal of Dental Education 14, no. 4 (2010): 193–202. 20946246 10.1111/j.1600-0579.2009.00609.x

[3] C. M. Serrano , M. G. Botelho , P. R. Wesselink , and J. M. Vervoorn , “Challenges in the Transition to Clinical Training in Dentistry: An ADEE Special Interest Group Initial Report,” European Journal of Dental Education 22, no. 3 (2018): e451–e457. 29396888 10.1111/eje.12324

[4] E. Monsef , X. Goodman , R. Patil , and S. N. White , “Dentists' Knowledge of Non‐Surgical Root Canal Treatment, a Systematic Review,” Journal of Dentistry 145 (2024): 104975. 38580057 10.1016/j.jdent.2024.104975

[5] A. E. Atherley , I. R. Hambleton , N. Unwin , C. George , P. M. Lashley , and C. G. Taylor , “Exploring the Transition of Undergraduate Medical Students Into a Clinical Clerkship Using Organizational Socialization Theory,” Perspectives on Medical Education 5, no. 2 (2016): 78–87. 26951164 10.1007/s40037-015-0241-5 PMC4839013

[6] C. Frese , D. Wolff , D. Saure , H. J. Staehle , and A. Schulte , “Psychosocial Impact, Perceived Stress and Learning Effect in Undergraduate Dental Students During Transition From Pre‐Clinical to Clinical Education,” European Journal of Dental Education 22, no. 3 (2018): e555–e563. 29635815 10.1111/eje.12352

[7] C. H. Grock , L. B. Luz , V. F. Oliveira , et al., “Experiences During the Execution of Emergency Endodontic Treatment and Levels of Anxiety in Dental Students,” European Journal of Dental Education 22, no. 4 (2018): e715–e723. 30079613 10.1111/eje.12385

[8] H. K. Yip and I. Barnes , “Learning in Dental Education,” European Journal of Dental Education 1, no. 2 (1997): 54–60. 9567901 10.1111/j.1600-0579.1997.tb00012.x

[9] R. De Moor , M. Hülsmann , L. L. Kirkevang , J. Tanalp , and J. Whitworth , “Undergraduate Curriculum Guidelines for Endodontology,” International Endodontic Journal 46, no. 12 (2013): 1105–1114. 24117830 10.1111/iej.12186

[10] American Association of Endodontists , “Guide to Clinical Endodontics,” (2020), https://www.aae.org/specialty/clinical‐resources/guide‐clinical‐endodontics/.

[11] J. Honey , C. D. Lynch , F. M. Burke , and A. S. M. Gilmour , “Ready for Practice? A Study of Confidence Levels of Final Year Dental Students at Cardiff University and University College Cork,” European Journal of Dental Education 15, no. 2 (2011): 98–103. 21492345 10.1111/j.1600-0579.2010.00646.x

[12] J. Puryer , S. Amin , and M. Turner , “Undergraduate Confidence When Undertaking Root Canal Treatment and Their Perception of the Quality of Their Endodontic Education,” Dentistry Journal 5, no. 1 (2016): 1. 29563408 10.3390/dj5010001 PMC5806992

[13] L. B. Luz , C. H. Grock , V. F. Oliveira , et al., “Self‐Reported Confidence and Anxiety Over Endodontic Procedures in Undergraduate Students‐Quantitative and Qualitative Study,” European Journal of Dental Education 23, no. 4 (2019): 482–490. 31373094 10.1111/eje.12456

[14] A. M. Hauser and D. M. Bowen , “Primer on Preclinical Instruction and Evaluation,” Journal of Dental Education 73, no. 3 (2009): 390–398. 19289728

[15] A. K. H. Pau and R. Croucher , “Emotional Intelligence and Perceived Stress in Dental Undergraduates,” Journal of Dental Education 67, no. 9 (2003): 1023–1028. 14518841

[16] M. B. Mirza , “Difficulties Encountered During Transition From Preclinical to Clinical Endodontics Among Salman Bin Abdul Aziz University Dental Students,” Journal of International Oral Health 7, no. Suppl 1 (2015): 22–27. PMC4516078 26225100

[17] M. O. S. Seijo , E. F. Ferreira , A. P. Ribeiro Sobrinho , S. M. Paiva , and R. C. Martins , “Learning Experience in Endodontics: Brazilian Students' Perceptions,” Journal of Dental Education 77, no. 5 (2013): 648–655. 23658412

[18] J. Tanalp , E. P. Güven , and I. Oktay , “Evaluation of Dental Students' Perception and Self‐Confidence Levels Regarding Endodontic Treatment,” European Journal of Dentistry 7, no. 2 (2013): 218–224. 24883030 10.4103/1305-7456.110189 PMC4023188

[19] H. A. Balto , “Attachment and Morphological Behavior of Human Periodontal Ligament Fibroblasts to Mineral Trioxide Aggregate: A Scanning Electron Microscope Study,” Journal of Endodontics 30, no. 1 (2004): 25–29. 14760903 10.1097/00004770-200401000-00005

[20] G. Wong , H. C. Apthorpe , K. Ruiz , and S. Nanayakkara , “Student‐To‐Student Dental Local Anesthetic Preclinical Training: Impact on Students' Confidence and Anxiety in Clinical Practice,” Journal of Dental Education 83, no. 1 (2019): 56–63. 30600250 10.21815/JDE.019.007

[21] G. I. Eleftheriadis and T. P. Lambrianidis , “Technical Quality of Root Canal Treatment and Detection of Iatrogenic Errors in an Undergraduate Dental Clinic,” International Endodontic Journal 38, no. 10 (2005): 725–734. 16164687 10.1111/j.1365-2591.2005.01008.x

[22] A. Donnelly , D. Coffey , and H. F. Duncan , “A Re‐Audit of the Technical Quality of Undergraduate Root Canal Treatment After the Introduction of New Technology and Teaching Practices,” International Endodontic Journal 50, no. 10 (2017): 941–950. 27917512 10.1111/iej.12727

[23] J. Davey , S. T. Bryant , and P. M. H. Dummer , “The Confidence of Undergraduate Dental Students When Performing Root Canal Treatment and Their Perception of the Quality of Endodontic Education,” European Journal of Dental Education 19, no. 4 (2015): 229–234. 25490882 10.1111/eje.12130

[24] M. R. G. Nassri , J. Carlik , C. R. N. da Silva , R. E. Okagawa , and S. Lin , “Critical Analysis of Artificial Teeth for Endodontic Teaching,” Journal of Applied Oral Science 16, no. 1 (2008): 43–49. 19089288 10.1590/S1678-77572008000100009 PMC4327279

[25] J. T. Nadler , R. Weston , and E. C. Voyles , “Stuck in the Middle: The Use and Interpretation of Mid‐Points in Items on Questionnaires,” Journal of General Psychology 142, no. 2 (2015): 71–89. 25832738 10.1080/00221309.2014.994590

[26] D. W. Chambers , “Toward a Competency‐Based Curriculum,” Journal of Dental Education 57, no. 11 (1993): 790–793. 8245288

[27] M. A. Stacey , M. V. Morgan , and C. Wright , “The Effect of Clinical Targets on Productivity and Perceptions of Clinical Competency,” Journal of Dental Education 62, no. 6 (1998): 409–414. 9698695

[28] D. Tuncer , N. Arhun , K. Yamanel , Ç. Çelik , and B. Dayangaç , “Dental Students' Ability to Assess Their Performance in a Preclinical Restorative Course: Comparison of Students' and Faculty Members' Assessments,” Journal of Dental Education 79, no. 6 (2015): 658–664. 26034030

[29] T. Gabbard and F. Romanelli , “The Accuracy of Health Professions Students' Self‐Assessments Compared to Objective Measures of Competence,” American Journal of Pharmaceutical Education 85, no. 4 (2021): 8405. 34283796 10.5688/ajpe8405 PMC8086612

[30] R. B. Kappler , K. B. De‐Paula , D. B. Barbisan , et al., “Preclinical Endodontics Teaching in Brazilian Dentistry Courses,” Rev ABENO 19, no. 2 (2019): 82–90.

[31] H. Al Raisi , P. M. H. Dummer , and M. E. Vianna , “How Is Endodontics Taught? A Survey to Evaluate Undergraduate Endodontic Teaching in Dental Schools Within the United Kingdom,” International Endodontic Journal 52, no. 7 (2019): 1077–1085. 30706491 10.1111/iej.13089

